# Discovering novel neuroactive drugs through high-throughput behavior-based chemical screening in the zebrafish

**DOI:** 10.3389/fphar.2014.00153

**Published:** 2014-07-24

**Authors:** Giancarlo Bruni, Parth Lakhani, David Kokel

**Affiliations:** Cardiovascular Research Center, Massachusetts General Hospital and Harvard Medical SchoolCharlestown, MA, USA

**Keywords:** behavior-based drug discovery, zebrafish, phenomics, antipsychotics, screening

## Abstract

Most neuroactive drugs were discovered through unexpected behavioral observations. Systematic behavioral screening is inefficient in most model organisms. But, automated technologies are enabling a new phase of discovery-based research in central nervous system (CNS) pharmacology. Researchers are using large-scale behavior-based chemical screens in zebrafish to discover compounds with new structures, targets, and functions. These compounds are powerful tools for understanding CNS signaling pathways. Substantial differences between human and zebrafish biology will make it difficult to translate these discoveries to clinical medicine. However, given the molecular genetic similarities between humans and zebrafish, it is likely that some of these compounds will have translational utility. We predict that the greatest new successes in CNS drug discovery will leverage many model systems, including *in vitro* assays, cells, rodents, and zebrafish.

## INTRODUCTION

At a recent course on neurotherapeutic drug discovery (sponsored by the National Institutes of Health) the keynote speaker joked that if he were trying to be rational, he would not be trying to discover neuroactive drugs. His point was that drug discovery is often much more empirical than rational. Someday, when researchers understand the biochemical mechanisms of psychiatric disease, it may be possible to discover neuroactive drugs based on rational therapeutic hypotheses. Until then, phenotypic assays provide an alternative approach. Behavior-based drug discovery is effective, but it needs to be more efficient.

Researchers can discover new drugs without understanding how they work. Neuroactive compounds including antipsychotics, antidepressants, and anxiolytics are among the top selling prescription drugs (Alonso et al., 2004; Gu et al., 2010; Alexander et al., 2011; Mojtabai and Olfson, 2014; Olfson et al., 2014). We know some details about how these drugs affect different neurotransmitter signaling pathways. But nobody really knows how these simple molecules change our moods, thoughts, and emotions. Target-based approaches to central nervous system (CNS) drug discovery have been largely unsuccessful (Paul et al., 2010). However, we can discover new drugs without understanding the details of how they work (Irwin, 1968; Tecott and Nestler, 2004). Historically, many neuroactive drugs were discovered despite totally incorrect therapeutic hypotheses (Sneader, 2005; Kokel and Peterson, 2008; Enna and Williams, 2009). So, although drug discovery and molecular understanding often go hand in hand—it is mostly in that order.

New technologies are changing how researchers use phenotypic assays to discover new drugs. Low throughput assays have limited the field with small sample sizes, narrow scope and limited hypothesis testing. Many key discoveries were made essentially by chance (Sneader, 2005; Enna and Williams, 2009). Now, high throughput assays are enabling a discovery-based approach that relies more on mathematical modeling and massive amounts of data (rather than theory and luck) to identify new drug leads (Schadt et al., 2009). Automated screening platforms do not need mechanistic theories to generate large data sets and identify correlations between compounds and phenotype. As a result, researchers can focus on discovering drugs and drug mechanisms as separate independent endeavors. Here, we review how this data-driven approach to behavioral phenomics is accelerating the pace of neuroactive drug discovery.

## HOW MANY NEUROACTIVE DRUGS ARE THERE?

“How many neuroactive drugs are there?” is a deceptively simple question that can be surprising difficult to answer. Neuroactive drugs are difficult to classify because relationships between compound structure, target and phenotype are often unclear and poorly understood. Structure-based classification is difficult because small structural changes can drastically alter a compound’s mechanism of action. Target-based classification is difficult because drug targets are often unknown. Even when *in vitro* targets are identified, their *in vivo* relevance is often unclear. One approach is to classify compounds based on behavioral phenotypes or medical utility. But most phenotype-based classifications are subjective and difficult to quantify. How do we know when a drug is an antipsychotic or an antidepressant (Maher et al., 2011)? There are no known molecular causes or biomarker-based diagnostics for most mental disorders (Javitt et al., 2008) and off-label prescriptions are common (Chouinard, 2006; Alexander et al., 2011). So exactly how many neuroactive drugs are there?

**FIGURE 1:**
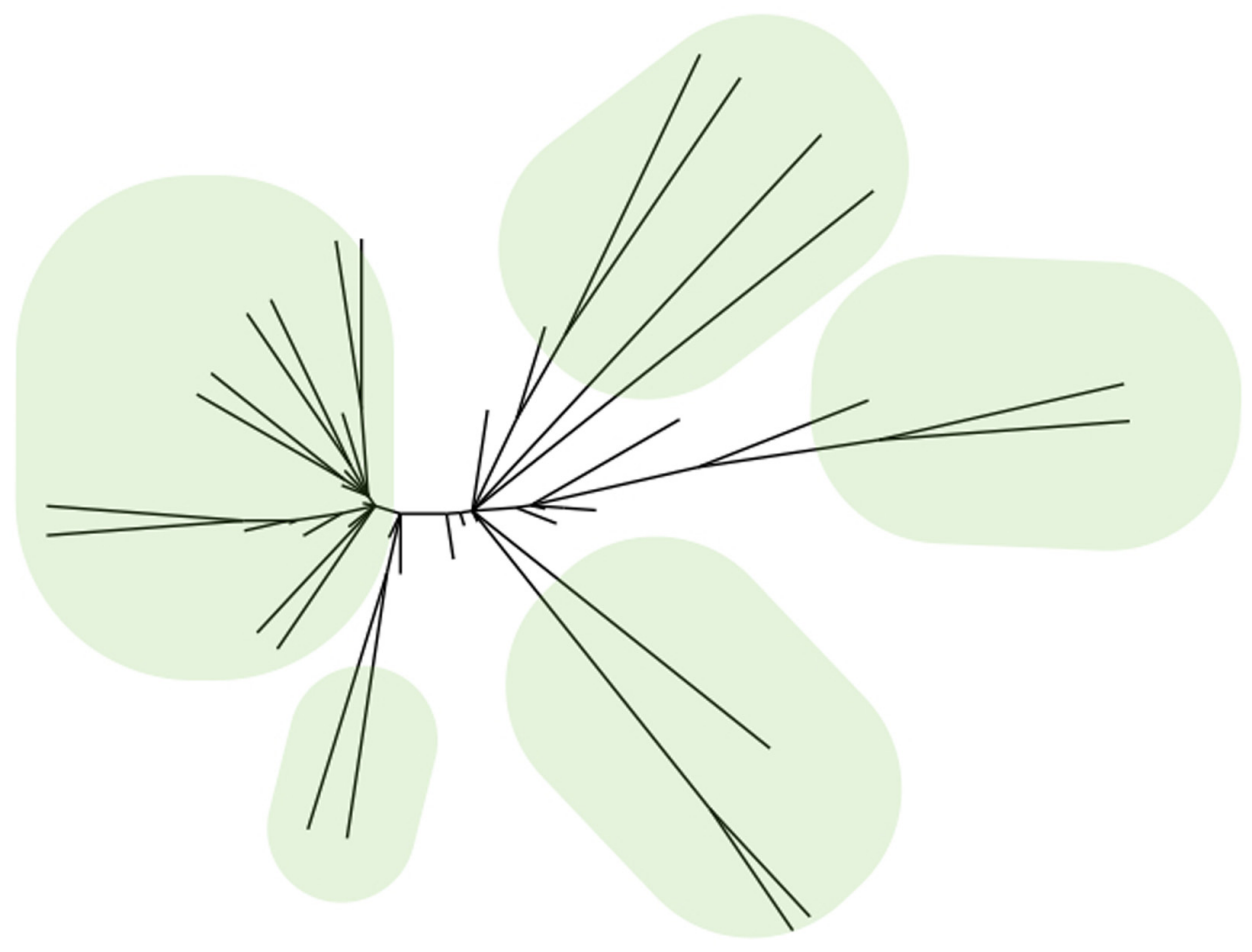
**Many drugs are structural analogs of each other.** Hierarchical cluster tree of 42 drugs labeled as “antipsychotic” in the Drugbank database. Five major families are indicated with ovals. Each family stems from a single prototype molecule.

Although the FDA lists thousands of antipsychotics, antidepressants and anxiolytics, most of these compounds fall into just a few structural classes. Consider the antipsychotics. Searching the FDALabel database for “antipsychotic” returns 1,325 hits, but most are mixtures and formulations of identical compounds (U.S. Food and Drug Administration, 2014). The same search in Drugbank returns 42 hits and most are close structural analogs of each other (Kokel and Peterson, 2008). Chemoinformatic algorithms cluster these compounds into a small number of structurally related families (Cao et al., 2008; Backman et al., 2011; **Figure 1**). Like antipsychotics, the antidepressants and anxiolytics show a similar pattern: There are many individual drugs, but most are structural analogs of a handful of prototypes. These data suggest that many drugs seem to discover themselves, due to the exploitation of prototype molecules (Sneader, 1996).

Most neuroactive drug prototypes were discovered during two broad time periods: pre-history and the mid-1900s. Both waves of discovery coincided with the availability of new chemical compounds alongside relatively widespread human and animal experimentation. The first wave of drugs, discovered in prehistoric times, were found by screening (ingesting) natural products in the environment. Compounds like morphine, alcohol, nicotine, and cocaine were identified based on their strange and unexpected behavioral phenotypes. The second wave of drugs, discovered in the mid-1900s, were found by screening synthetic compounds. These drugs, including the first modern anxiolytics, antipsychotics and antidepressants, were also discovered based on unexpected behavioral phenotypes.

**FIGURE 2:**
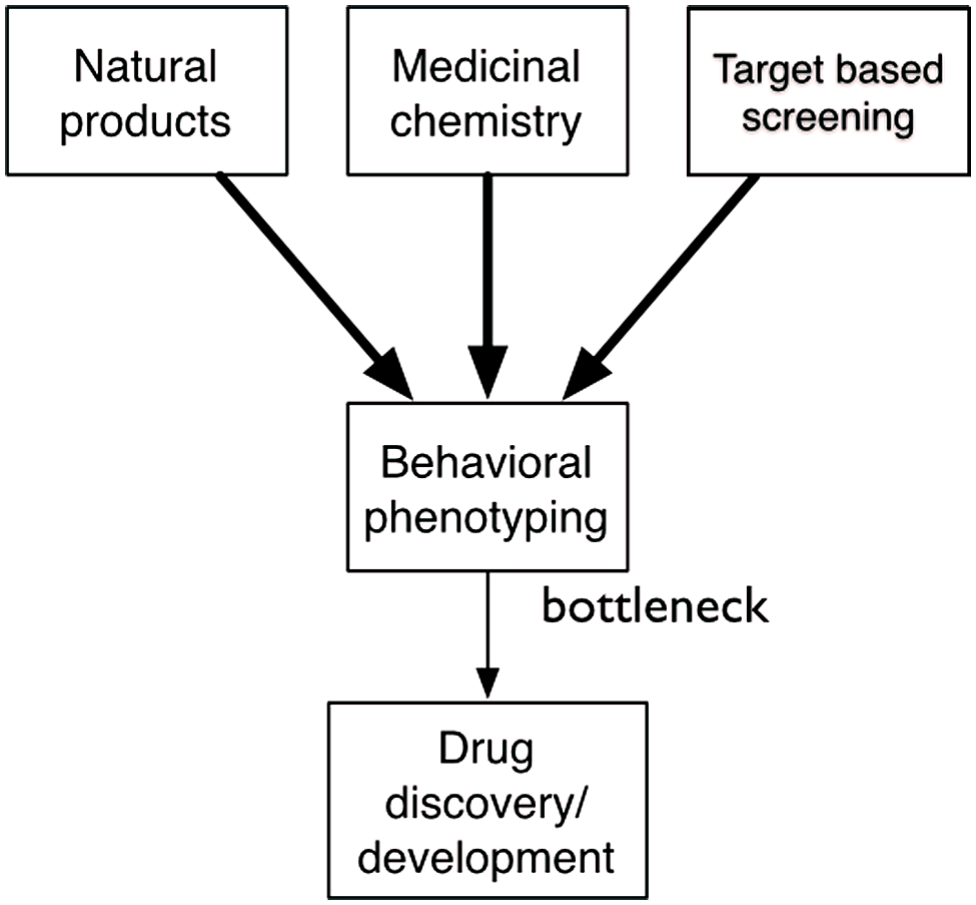
**Behavioral phenotyping is a key bottleneck in drug discovery.** Although there is an abundance of small molecules from nature, medicinal chemistry, and target-based screening only a very small number are ever tested in behavioral assays, limiting the drug discovery process.

Behavioral phenotyping is an essential part of drug discovery, but it is also the bottleneck (**Figure 2**). Prototype discovery often starts with the observation of an unexpected behavioral phenotype. Once a prototype has been identified, medicinal chemists generate structural analogs that themselves often have unexpected phenotypes. Researchers use these compounds to test therapeutic hypotheses and search for mechanistic understanding. When molecular targets are identified, researchers search for new ligands that trigger a new round of behavioral phenotyping. In target-based approaches, behavioral phenotyping is deferred until later in the process. Ultimately, the final step of determining efficacy in humans is also a matter of behavioral phenotyping. The process is incredibly effective and has generated most drugs that we use today. Medicinal chemistry can efficiently generate thousands of structural analogs; technologies for *in vitro* screening are ultra high-throughput. But, decades-old approaches to behavioral phenotyping throttle the drug discovery engine.

## WHAT DO ZEBRAFISH DO?

The zebrafish model system enables researchers to combine complex behavioral phenotyping with high-throughput chemical screening. Like humans, fish are vertebrate animals with complex brains and behaviors. But unlike humans, fish are small enough to fit in 96-well plates and they easily absorb compounds dissolved in the water. These features make zebrafish uniquely well suited for phenotype-based neuroactive drug discovery and enable researchers to scale complex behavioral assays to high-throughput formats.

A frequently asked question about behavior-based drug discovery in zebrafish is “What do zebrafish do?” Personality disorders, depression, and anxiety seem like some of the most complex phenotypes imaginable. Fish do not suffer from these feelings the same way that people do. So, the idea of using fish to discover neuroactive drugs can seem counterintuitive. We tend to think about zebrafish behavior in two different ways: where as some fish behaviors resemble some human behaviors, many others lack obvious human correlates.

Anthropomorphic assays in fish are very powerful. We immediately empathize with familiar behaviors and their circuitry and mechanisms are likely to be relatively well conserved. For example, researchers use circadian cycles in zebrafish locomotor activity to study mechanism that control sleep behaviors in humans (Prober et al., 2006; Zhdanova, 2006; Yokogawa et al., 2007; Rihel et al., 2010). Researchers also use zebrafish behavior to study pain (Prober et al., 2008), fear (Speedie and Gerlai, 2008; Agetsuma et al., 2010; Mathuru et al., 2012), learned helplessness (Lee et al., 2010), feeding (Gahtan et al., 2005; Del Bene et al., 2010; Bianco et al., 2011), courtship (Darrow and Harris, 2004), learning (Valente et al., 2012), vision (Emran et al., 2007), hearing (Gleason et al., 2009), touch (Low et al., 2010, 2011, 2012), social interactions (Pérez-Escudero and de Polavieja, 2011; Mahabir et al., 2013; Qin et al., 2014), anxiety (Stewart et al., 2012), and decision making (Arganda et al., 2012).

By contrast, it is more difficult to empathize with fish-specific phenotypes. The practical implications of understanding fundamental fish behaviors are not always obvious. And it is easy focus on their differences rather then their similarities. Nevertheless, neuroactive drugs affect fish behavior in specific and reproducible ways via conserved molecular mechanisms. Adult zebrafish, differentially change their swimming and three-dimensional tank diving behaviors in response to many neuroactive compounds (Cachat et al., 2011; Grossman et al., 2011; Stewart et al., 2011; Kyzar et al., 2012a, 2013; Williams et al., 2012; Robinson et al., 2013; Stewart and Kalueff, 2014). In larvae, fish specific behaviors like spontaneous swimming (Wyart et al., 2009), the optokinetic reflex (Emran et al., 2007) and photomotor response (Kokel et al., 2013b) can be used to understand neuronal signaling, rapidly identify novel neuroactive compounds and predict their mechanisms of action (Kokel et al., 2010; Rihel et al., 2010). The key challenge is learning how to decode complex patterns of behavior to understand which pathways are being modulated—and how they may affect human health.

## A DISCOVERY-BASED APPROACH

Some of the most exciting developments in behavioral phenomics are coming from two very different models: humans and zebrafish. Compared to other animals, human behaviors are probably the most complex, variable and challenging to measure. So it is somewhat surprising that human behavioral phenomics is advancing so rapidly. One reason is that substantial investments by internet technology companies have increased the scale of digital record keeping and chemobehavioral phenotyping. Large medical databases link people’s genotype, phenotype and prescription drug records. Researchers are mining these databases to identify unanticipated drug side effects and repurpose drugs for new indications (Dudley et al., 2010).

Human behavioral phenomics is a powerful way to approach drug repurposing, but it cannot be used for chemical screening. Governmental and institutional regulations limit large-scale human studies to compounds that are already approved by the FDA (thankfully). Researchers will need other model organisms, like zebrafish, to systematically discover new molecular entities. Until recently, tools for high throughput behavioral phenotyping were unavailable. But new technologies are changing the drug discovery landscape.

## AUTOMATED SOLUTIONS

Automated technologies are making behavior-based chemical screening in zebrafish a more effective, efficient and systematic way to discover neuroactive compounds. Three aspects of automation are changing the field of behavior-based drug screening: robotics, analytics, and academic industrial collaboration. These changes are a small part of larger global trends in computing technology. As sophisticated processors, programming languages, and rapid prototyping tools become more accessible, individual scientists and small academic laboratories are innovating alongside larger biotechnology and pharmaceutical companies.

Robotic solutions are growing to meet nearly every early step of the screening process including fish breeding, sorting, and phenotyping. Robotic aquaculture racks automate feeding cycles and monitor water quality. Specialized breeding tanks produce thousands of synchronized embryos (Adatto et al., 2011). Flow cytometry platforms sort zebrafish into 96-well plates. And imaging platforms automate morphological and behavioral phenotyping (Burgess and Granato, 2007a; Pardo-Martin et al., 2010a, 2013; Ahrens et al., 2012; Engert, 2012; Wittmann et al., 2012). For example, researchers have developed an elegant and powerful (freely available) software package, FLOTE, for automated tracking of precise kinematic events in larval zebrafish (Burgess and Granato, 2007a). The software has already been used to analyze startle modulation, light adaptation, and navigation (Burgess and Granato, 2007a,b; Burgess et al., 2009, 2010; Jain et al., 2011; Fernandes et al., 2012). The software has also been used to find compounds that modulate memory formation in larval zebrafish (Wolman et al., 2011). Although not yet used for drug screening, recent advances in whole-brain functional imaging record patterns of firing activity of individual cells in large populations of neurons (Ahrens et al., 2012, 2013; Kokel et al., 2013b; Muto et al., 2013; Satou et al., 2013; Portugues et al., 2014) and will likely add massive amounts data to the behavioral pharmacology field. As behavioral datasets grow, researchers are applying new analytical approaches to explore, organize, and discover correlations between phenotypic patterns and compound treatments.

Academic-industrial partnerships are improving zebrafish phenotyping and phenotype-based approaches to drug discovery. The innovations flow both ways, from academia to industry and industry to academia. Acquifer (http://www.acquifer.de), a new biotech company with roots in academic automated zebrafish phenotyping, is developing network platforms for managing huge amounts of data from zebrafish phenotypic screens. Commercial imaging platforms, like the Vertebrate Automated Screening Technology marketed is based on academic innovations (Pardo-Martin et al., 2010b, 2013; Chang et al., 2012). When equipment is too expensive, academic bioinstrumentation laboratories are working to develop more affordable do-it-yourself kits (Alper, 2009; Marzullo and Gage, 2012). As sophisticated rapid prototyping tools become more accessible (like 3D printers, open source programming languages, and cheap microcontrollers) the pace of innovation is accelerating.

## SCALING BEHAVIORAL DATABASES INTO CONNECTIVITY MAPS

Today, database-linked tools for analyzing gene expression data and behavioral data look very different. Behavioral databases tend to be designed for finding and summarizing data via search field descriptors like compound name, genes name and strain name. For example, the Zebrafish Neurophenome Database (ZND) is a publically available database designed to provide a comprehensive resource of neurobehavioral phenotypes in adult zebrafish (Green et al., 2012; Kyzar et al., 2012b; Kalueff et al., 2013). To search the ZND, a researcher uses drop-down fields to select investigator and drugs of interest to experimental results and drug effects that are often presented as textual descriptions. Similarly, large-scale mouse phenotyping projects like the Mouse Phenome Database (MPD) at The Jackson Laboratory allow users to find, visualize and analyze mouse behavioral phenotypes across different strains and conditions (Maddatu et al., 2012). The MPD stores a large amount of standardized, quantifiable and comparable data (like weight and grip strength). The MPD also provides a variety of tools to analyze results (including tools to find strains that best fit phenotypic criteria). But, as phenotypic databases grow ever larger, they will enable more complex data-driven queries.

Given sufficiently rich behavioral phenotyping, it should be possible to build a connectivity map to systematically identify neuroactive compounds and sort them into phenotypic classes. For example, the Connectivity Map is designed to use gene expression data as a discovery framework by allowing researchers to use gene expression signatures to query the data for closely related perturbagens (Lamb et al., 2006; Lamb, 2007). As a result, one can use the data itself to identify correlations, perform cluster analyses and identify outliers. Analyses that were originally developed for applications like speech recognition and social networking can just as easily be applied to analyzing zebrafish phenotypes. And these analyses allow new questions about large diverse data sets. We imagine that someday soon, it may be possible to query large behavioral databases with BLAST-like and speech recognition tools. This could allow researchers to identify all compounds with similar behavioral phenotypes, link genetic mutants to small molecule treatments and identify new treatments with totally novel phenotypes. Will it be possible to identify just the right pattern of fish behaviors to accurately identify drugs with complex activities in humans (like antipsychotics and antidepressants)? Future studies may provide the answer.

## WHAT ARE WE LIKELY TO FIND?

Given that so few compounds have been tested in animals, large-scale behavioral screens are almost guaranteed to identify new neuroactive compounds. These studies will provide high-resolution maps of how small molecules affect the brain and behavior. But what kinds of compounds are likely to be discovered? Are we really likely to identify new compounds with new mechanisms of action? Or just more of the same kinds of drugs we already have? The data supports both arguments.

On the one hand, one could argue that behavior-based drug screening has been saturated: Multiple classes of antipsychotics, antidepressants, and anxiolytics have already been identified. One possibility is that the low throughput non-systematic approaches employed in the past have already identified all the neuroactive drugs worth discovering. Alternatively, it is interesting to speculate that compounds with antipsychotic, antidepressant and anxiolytic effects may be relatively common. If so, large-scale screens would likely identify a variety of new psychotropic drug prototypes with a range of phenotypic and mechanistic profiles, including totally new structures, mechanisms, and phenotypes.

Large zebrafish behavior-based chemical screens are already identifying a variety of new compounds. Some of first neuroactive compounds to be discovered in zebrafish, str1, and str2, were novel acetylcholinesterase inhibitors (Kokel et al., 2010). These compounds were new molecular entities, but they were not first in class compounds. These compounds may provide modest advantages over current treatment options. But identifying novel structures with novel targets and mechanisms would have a greater impact. One potential way to identify compounds with novel mechanisms is to identify compounds that cause outlier phenotypes in behavioral databases. If one compound in ten thousand causes a unique behavioral phenotype, this suggests it may be working through a new (and rare) mechanism of action. For example, a new kind of light controllable rapidly reversible TrpA1 ligand, optovin, was recently discovered in just this way (Kokel et al., 2013a). Several novel light activated molecules have been developed using zebrafish behavioral readouts (Szobota et al., 2007; Janovjak et al., 2010; Levitz et al., 2013). This suggests that truly novel compounds are waiting to be found, if only we use the right methods to look for them.

## MODIFIER SCREENS: CHEMICAL AND GENETIC MODELS

Although wild-type phenotypes may be useful for identifying certain compounds, we can also use chemical and genetic tools to model specific disease states. These disease models combine the advantages of unbiased phenotypic screening with readouts that are specifically designed to target certain kinds of compounds. In one recent example, researchers identified a zebrafish mutant (in the Scn1a gene) and then used this model to screen for potential treatments for Dravet syndrome (caused by mutations in the homologous human gene; Baraban et al., 2013). These researchers identified an FDA approved compound that suppressed the fish phenotype, suggesting that the approach may be a powerful way to identify therapeutics for this specific disorder. This work elegantly illustrates the potential for genetic models in zebrafish to identify desperately needed targeted therapeutics with potential utility in humans. One can imagine many variations on this theme. CRISP-Cas technology is revolutionizing zebrafish researchers’ ability to efficiently generate knockout and knock-in models (Hwang et al., 2013a,b; Auer et al., 2014). Transgenic overexpression models phenocopy aspects of neurodegenerative and other dominant diseases (Bai et al., 2007; Olson et al., 2010). And, due to the ease of chemical manipulations, researchers have run large-scale modifier screens in chemically treated disease models (Baraban et al., 2005).

## WHOLE ORGANISM PHENOTYPING: BLOOD–BRAIN BARRIER AND TOXICOLOGY

Researchers can expand phenotypic readouts to encompass almost any aspect the organism including blood–brain barrier (BBB), toxicity, and cardiovascular readouts. Larval zebrafish develop a functional BBB with size exclusion and transport pumps including those that are similar to mammals (Jeong et al., 2008). So researchers have some reason to believe compounds with CNS activity in fish may also penetrate the BBB in mammals. Similarly, potentially toxic compounds can be screened for unwanted and unexpected toxic or cardiovascular side effects. One could potentially capture data on zebrafish development, behavior and heart rate simultaneously in a high-throughput and automated fashion. Because researchers can apply diverse phenotyping assays, zebrafish are an exciting model for toxicology in addition to drug discovery.

## THE CHALLENGE OF TRANSLATING FROM FISH TO HUMANS

Despite the power of new technologies, there are substantial fundamental challenges to translating CNS drug discovery from fish to humans. Most investigational new drugs fail when they are finally tested (for efficacy) in humans (Paul et al., 2010). There are many reasons why preclinical predictions from any model system would fail to translate, but lack of rigor should not be one of them.

Inefficient animal studies contribute to publication bias, decrease scientific rigor, and limit the drug discovery process. Compared to zebrafish, studies in larger animals, like mice, are relatively expensive and require substantial amounts of test compounds. Due to these costs, some large-animal studies tend to be underpowered, which contributes to irreproducible results (Landis et al., 2012). Zebrafish enable a level of rigor and reproducibility that can be difficult to achieve in larger model organisms, simply because the assays can be easily reproduced on larger scales. Hypotheses can be tested on thousands of animals, rather than just a handful, at small cost in time and other resources. For example, treating a single mouse (at 10 mg/kg) requires approximately 100X more compound than is needed to treat a well of zebrafish (at 10 μM). When researchers increase sample size it becomes easier to find true signals amongst the noise. However, even if new compounds can be discovered with reproducible effects on zebrafish behavior, substantial challenges remain to translate these discoveries for improving human health.

**FIGURE 3:**
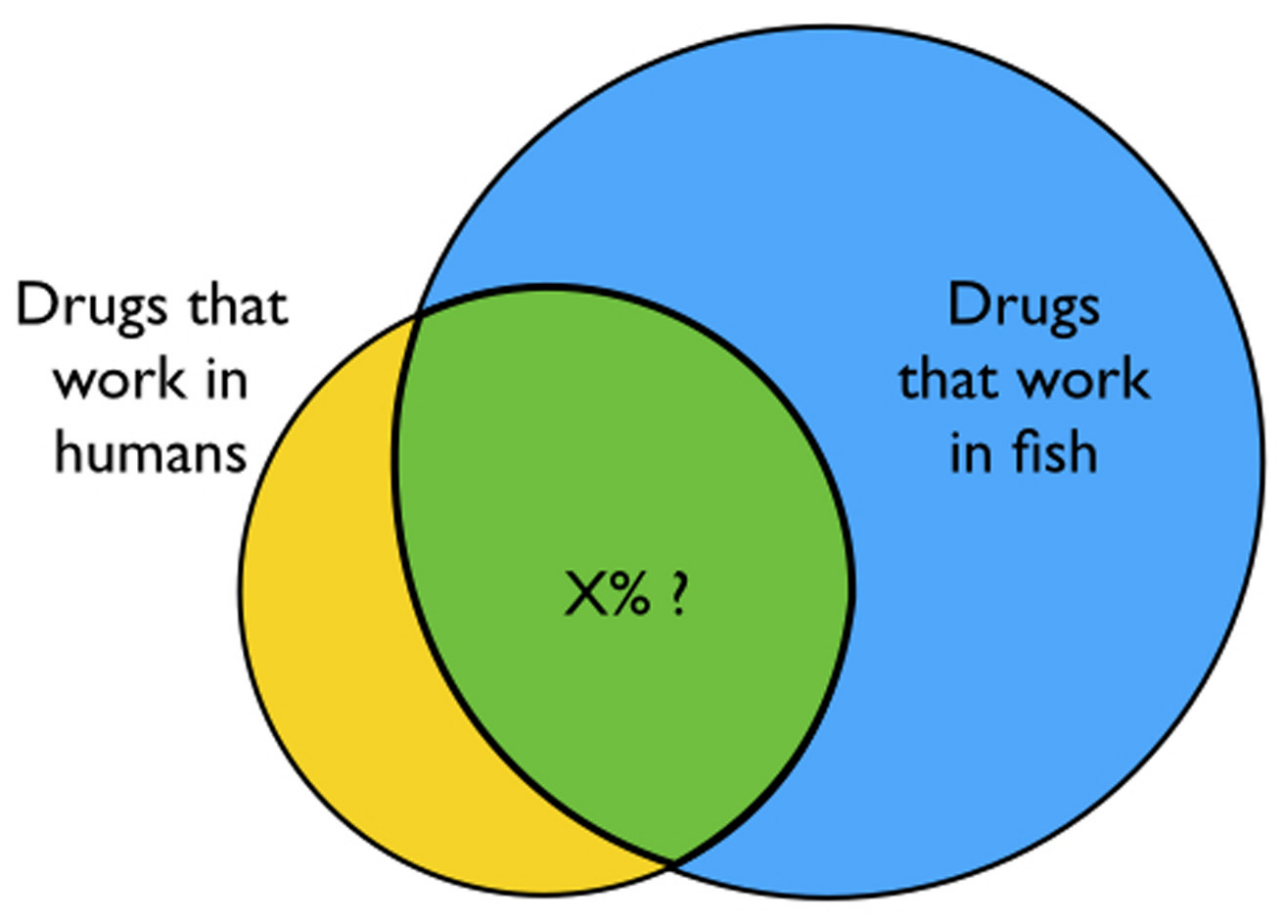
**How many drugs work in both humans and zebrafish? Not all drugs that work in zebrafish will also work in humans.** Because so many more compounds can be screened in zebrafish, it is likely that some will translate to the clinic. However, the precise level of overlap (indicated by X) is difficult to predict.

Many compounds work in humans, many work in zebrafish, and some fraction is likely to work in both–although the exact level of overlap is difficult to predict (**Figure 3**). Humans and zebrafish are closely related (Howe et al., 2013), but there are many differences at the phenotypic, neuronal network, and molecular levels. When a new bioactive compound is first discovered in zebrafish, it will be difficult to predict its potential therapeutic utility in humans. Many compounds that appear to work well in mice and other animal models subsequently fail to translate to humans. The same will surely be true of zebrafish. The problem is especially relevant in neuropharmacology, where CNS disorders are poorly understood and difficult to model. Despite the challenges, in the upcoming years we are likely to see at least a few compounds identified in zebrafish screens translate from bench to bedside.

## Conflict of Interest Statement

The authors declare that the research was conducted in the absence of any commercial or financial relationships that could be construed as a potential conflict of interest.

## References

[B1] Adatto I. Lawrence C. Thompson M. Zon L. I. (2011). A new system for the rapid collection of large numbers of developmentally staged zebrafish embryos. *PLoS ONE* 6:e21715 10.1371/journal.pone.0021715 PMC3126846 21738776

[B2] Agetsuma M. Aizawa H. Aoki T. Nakayama R. Takahoko M. Goto M. (2010). The habenula is crucial for experience-dependent modification of fear responses in zebrafish. *Nat. Neurosci.* 13 1354–1356 10.1038/nn.2654 20935642

[B3] Ahrens M. B. Li J. M. Orger M. B. Robson D. N. Schier A. F. Engert F. (2012). Brain-wide neuronal dynamics during motor adaptation in zebrafish. *Nature* 485 471–477 10.1038/nature11057 22622571 PMC3618960

[B4] Ahrens M. B. Orger M. B. Robson D. N. Li J. M. Keller P. J. (2013). Whole-brain functional imaging at cellular resolution using light-sheet microscopy. *Nat. Methods* 10 413–420 10.1038/nmeth.2434 23524393

[B5] Alexander G. C. Gallagher S. A. Mascola A. Moloney R. M. Stafford R. S. (2011). Increasing off-label use of antipsychotic medications in the United States, 1995–2008. *Pharmacoepidemiol. Drug Saf.* 20 177–184 10.1002/pds.2082 21254289 PMC3069498

[B6] Alonso J. Angermeyer M. C. Bernert S. Bruffaerts R. Brugha T. S. Bryson H. (2004). Psychotropic drug utilization in Europe: results from the European Study of the Epidemiology of Mental Disorders (ESEMeD) project. *Acta Psychiatr. Scand. Suppl.* 109 55–64 10.1111/j.1600-0047.2004.00325.x 15128388

[B7] Alper J. (2009). Biotech in the basement. *Nat. Biotechnol.* 27 1077–1078 10.1038/nbt1209-1077 20010575

[B8] Arganda S. Pérez-Escudero A. de Polavieja G. G. (2012). A common rule for decision making in animal collectives across species. *Proc. Natl. Acad. Sci. U.S.A.* 109 20508–20513 10.1073/pnas.1210664109 23197836 PMC3528575

[B9] Auer T. O. Duroure K. De Cian A. Concordet J.-P. Del Bene F. (2014). Highly efficient CRISPR/Cas9-mediated knock-in in zebrafish by homology-independent DNA repair. *Genome Res.* 24 142–153 10.1101/gr.161638.113 24179142 PMC3875856

[B10] Backman T. W. H. Cao Y. Girke T. (2011). ChemMine tools: an online service for analyzing and clustering small molecules. *Nucleic Acids Res.* 39 W486–W491 10.1093/nar/gkr320 21576229 PMC3125754

[B11] Bai Q. Garver J. A. Hukriede N. A. Burton E. A. (2007). Generation of a transgenic zebrafish model of Tauopathy using a novel promoter element derived from the zebrafish *eno2* gene. *Nucleic Acids Res.* 35 6501–6516 10.1093/nar/gkm608 17897967 PMC2095798

[B12] Baraban S. C. Dinday M. T. Hortopan G. A. (2013). Drug screening in *Scn1a* zebrafish mutant identifies clemizole as a potential Dravet syndrome treatment. *Nat. Commun.* 4 2410 10.1038/ncomms3410 PMC3891590 24002024

[B13] Baraban S. C. Taylor M. R. Castro P. A. Baier H. (2005). Pentylenetetrazole induced changes in zebrafish behavior, neural activity and c-fos expression. *Neuroscience* 131 759–768 10.1016/j.neuroscience.2004.11.031 15730879

[B14] Bianco I. H. Kampff A. R. Engert F. (2011). Prey capture behavior evoked by simple visual stimuli in larval zebrafish. *Front. Syst. Neurosci.* 5:101 10.3389/fnsys.2011.00101 PMC3240898 22203793

[B15] Burgess H. A. Granato M. (2007a). Modulation of locomotor activity in larval zebrafish during light adaptation. *J. Exp. Biol.* 210 2526–2539 10.1242/jeb.003939 17601957

[B16] Burgess H. A. Granato M. (2007b). Sensorimotor gating in larval zebrafish. *J. Neurosci.* 27 4984–4994 10.1523/JNEUROSCI.0615-07.2007 17475807 PMC6672105

[B17] Burgess H. A. Johnson S. L. Granato M. (2009). Unidirectional startle responses and disrupted left-right co-ordination of motor behaviors in robo3 mutant zebrafish. *Genes Brain Behav.* 8 500–511 10.1111/j.1601-183X.2009.00499.x 19496826 PMC2752477

[B18] Burgess H. A. Schoch H. Granato M. (2010). Distinct retinal pathways drive spatial orientation behaviors in zebrafish navigation. *Curr. Biol.* 20 381–386 10.1016/j.cub.2010.01.022 20153194 PMC3412192

[B19] Cachat J. Stewart A. Utterback E. Hart P. Gaikwad S. Wong K. (2011). Three-dimensional neurophenotyping of adult zebrafish behavior. *PLoS ONE* 6:e17597 10.1371/journal.pone.0017597 PMC3049776 21408171

[B20] Cao Y. Charisi A. Cheng L.-C. Jiang T. Girke T. (2008). ChemmineR: a compound mining framework for R. *Bioinformatics* 24 1733–1734 10.1093/bioinformatics/btn307 18596077 PMC2638865

[B21] Chang T.-Y. Pardo-Martin C. Allalou A. Wählby C. Yanik M. F. (2012). Fully automated cellular-resolution vertebrate screening platform with parallel animal processing. *Lab Chip* 12 711–716 10.1039/c1lc20849g 22159032 PMC3415711

[B22] Chouinard G. (2006). The search for new off-label indications for antidepressant, antianxiety, antipsychotic and anticonvulsant drugs. *J. Psychiatry Neurosci.* 31 168–176 16699602 PMC1449873

[B23] Darrow K. O. Harris W. A. (2004). Characterization and development of courtship in zebrafish, *Danio rerio*. *Zebrafish* 1 40–45 10.1089/154585404774101662 18248204

[B24] Del Bene F. Wyart C. Robles E. Tran A. Looger L. Scott E. K. (2010). Filtering of visual information in the tectum by an identified neural circuit. *Science* 330 669–673 10.1126/science.1192949 21030657 PMC3243732

[B25] Dudley J. T. Schadt E. Sirota M. Butte A. J. Ashley E. (2010). Drug discovery in a multidimensional world: systems, patterns, and networks. *J. Cardiovasc. Transl. Res.* 3 438–447 10.1007/s12265-010-9214-6 20677029

[B26] Emran F. Rihel J. Adolph A. R. Wong K. Y. Kraves S. Dowling J. E. (2007). OFF ganglion cells cannot drive the optokinetic reflex in zebrafish. *Proc. Natl. Acad. Sci. U.S.A.* 104 19126–19131 10.1073/pnas.0709337104 18025459 PMC2141919

[B27] Engert F. (2012). Fish in the matrix: motor learning in a virtual world. *Front. Neural Circuits* 6:125 10.3389/fncir.2012.00125 PMC3555039 23355810

[B28] Enna S. J. Williams M. (2009). Challenges in the search for drugs to treat central nervous system disorders. *J. Pharmacol. Exp. Ther.* 329 404–411 10.1124/jpet.108.143420 19182069

[B29] Fernandes A. M. Fero K. Arrenberg A. B. Bergeron S. A. Driever W. Burgess H. A. (2012). Deep brain photoreceptors control light-seeking behavior in zebrafish larvae. *Curr. Biol.* 22 2042–2047 10.1016/j.cub.2012.08.016 23000151 PMC3494761

[B30] Gahtan E. Tanger P. Baier H. (2005). Visual prey capture in larval zebrafish is controlled by identified reticulospinal neurons downstream of the tectum. *J. Neurosci.* 25 9294–9303 10.1523/JNEUROSCI.2678-05.2005 16207889 PMC6725764

[B31] Gleason M. R. Nagiela A. Jametb S. Vologodskaiaa M. López-Schiera H. Hudspetha A. J. (2009). The transmembrane inner ear (Tmie) protein is essential for normal hearing and balance in the zebrafish. *Proc. Natl. Acad. Sci. U.S.A.* 106 21347–21352 10.1073/pnas.0911632106 19934034 PMC2781060

[B32] Green J. Collins C. Kyzar E. J. Pham M. Roth A. Gaikwad S. (2012). Automated high-throughput neurophenotyping of zebrafish social behavior. *J. Neurosci. Methods* 210 266–271 10.1016/j.jneumeth.2012.07.017 22884772

[B33] Grossman L. Stewart A. Gaikwad S. Utterback E. Wu N. Dileo J. (2011). Effects of piracetam on behavior and memory in adult zebrafish. *Brain Res. Bull.* 85 58–63 10.1016/j.brainresbull.2011.02.008 21371538

[B34] Gu Q. Dillon C. F. Burt V. L. (2010). Prescription drug use continues to increase: U.S. prescription drug data for 2007–2008. *NCHS Data Brief* 42 1–8 20854747

[B35] Howe K. Clark M. D. Torroja C. F. Torrance J. Berthelot C. Muffato M. (2013). The zebrafish reference genome sequence and its relationship to the human genome. *Nature* 496 498–503 10.1038/nature12111 23594743 PMC3703927

[B36] Hwang W. Y. Fu Y. Reyon D. Maeder M. L. Kaini P. Sander J. D. (2013a). Heritable and precise zebrafish genome editing using a CRISPR-Cas system. *PLoS ONE* 8:e68708 10.1371/journal.pone.0068708 PMC3706373 23874735

[B37] Hwang W. Y. Fu Y. Reyon D. Maeder M. L. Tsai S. Q. Sander J. D. (2013b). Efficient genome editing in zebrafish using a CRISPR-Cas system. *Nat. Biotechnol.* 31 227–229 10.1038/nbt.2501 23360964 PMC3686313

[B38] Irwin S. (1968). Comprehensive observational assessment: Ia. A systematic, quantitative procedure for assessing the behavioral and physiologic state of the mouse. *Psychopharmacology (Berl.)* 13 222–257 10.1007/BF00401402 5679627

[B39] Jain R. A. Wolman M. A. Schmidt L. A. Burgess H. A. Granato M. (2011). Molecular-genetic mapping of zebrafish mutants with variable phenotypic penetrance. *PLoS ONE* 6:e26510 10.1371/journal.pone.0026510 PMC3198425 22039502

[B40] Janovjak H. Szobota S. Wyart C. Trauner D. Isacoff E. Y. (2010). A light-gated, potassium-selective glutamate receptor for the optical inhibition of neuronal firing. *Nat. Neurosci.* 13 1027–1032 10.1038/nn.2589 20581843 PMC2915903

[B41] Javitt D. C. Spencer K. M. Thaker G. K. Winterer G. Hajós M. (2008). Neurophysiological biomarkers for drug development in schizophrenia. *Nat. Rev. Drug Discov.* 7 68–83 10.1038/nrd2463 18064038 PMC2753449

[B42] Jeong J.-Y. Kwon H. B. Ahn J. C. Kang D. Kwon S. H. Park J. A. (2008). Functional and developmental analysis of the blood-brain barrier in zebrafish. *Brain Res. Bull.* 75 619–628 10.1016/j.brainresbull.2007.10.043 18355638

[B43] Kalueff A. V. Gebhardt M. Stewart A. M. Cachat J. M. Brimmer M. Chawla J. S. (2013). Towards a comprehensive catalog of zebrafish behavior 1.0 and beyond. *Zebrafish* 10 70–86 10.1089/zeb.2012.0861 23590400 PMC3629777

[B44] Kokel D. Bryan J. Laggner C. White R. Cheung C. Y. Mateus R. (2010). Rapid behavior-based identification of neuroactive small molecules in the zebrafish. *Nat. Chem. Biol.* 6 231–237 10.1038/nchembio.307 20081854 PMC2834185

[B45] Kokel D. Cheung C. Y. Mills R. Coutinho-Budd J. Huang L. Setola V. (2013a). Photochemical activation of TRPA1 channels in neurons and animals. *Nat. Chem. Biol.* 9 257–263 10.1038/nchembio.1183 23396078 PMC3604056

[B46] Kokel D. Dunn T. W. Ahrens M. B. Alshut R. Cheung C. Y. Saint-Amant L. (2013b). Identification of nonvisual photomotor response cells in the vertebrate hindbrain. *J. Neurosci.* 33 3834–3843 10.1523/JNEUROSCI.3689-12.2013 23447595 PMC3600642

[B47] Kokel D. Peterson R. T. (2008). Chemobehavioural phenomics and behaviour-based psychiatric drug discovery in the zebrafish. *Brief. Funct. Genomic. Proteomic.* 7 483–490 10.1093/bfgp/eln040 18784194 PMC2722257

[B48] Kyzar E. J. Collins C. Gaikwad S. Green J. Roth A. Monnig L. (2012a). Effects of hallucinogenic agents mescaline and phencyclidine on zebrafish behavior and physiology. *Prog. Neuropsychopharmacol. Biol. Psychiatry* 37 194–202 10.1016/j.pnpbp.2012.01.003 22251567 PMC3294104

[B49] Kyzar E. Zapolsky I. Green J. Gaikwad S. Pham M. Collins C. (2012b). The Zebrafish Neurophenome Database (ZND): a dynamic open-access resource for zebrafish neurophenotypic data. *Zebrafish* 9 8–14 10.1089/zeb.2011.0725 22171801

[B50] Kyzar E. Stewart A. M. Landsman S. Collins C. Gebhardt M. Robinson K. (2013). Behavioral effects of bidirectional modulators of brain monoamines reserpine and d-amphetamine in zebrafish. *Brain Res.* 1527 108–116 10.1016/j.brainres.2013.06.033 23827499 PMC3865859

[B51] Lamb J. (2007). The Connectivity Map: a new tool for biomedical research. *Nat. Rev. Cancer* 7 54–60 10.1038/nrc2044 17186018

[B52] Lamb J. Crawford E. D. Peck D. Modell J. W. Blat I. C. Wrobel M. J. (2006). The Connectivity Map: using gene-expression signatures to connect small molecules, genes, and disease. *Science* 313 1929–1935 10.1126/science.1132939 17008526

[B53] Landis S. C. Amara S. G. Asadullah K. Austin C. P. Blumenstein R. Bradley E. W. (2012). A call for transparent reporting to optimize the predictive value of preclinical research. *Nature* 490 187–191 10.1038/nature11556 23060188 PMC3511845

[B54] Lee A. Mathuru A. S. Teh C. Kibat C. Korzh V. Penney T. B. (2010). The habenula prevents helpless behavior in larval zebrafish. *Curr. Biol.* 20 2211–2216 10.1016/j.cub.2010.11.025 21145744

[B55] Levitz J. Pantoja C. Gaub B. Janovjak H. Reiner A. Hoagland A. (2013). Optical control of metabotropic glutamate receptors. *Nat. Neurosci.* 16 507–516 10.1038/nn.3346 23455609 PMC3681425

[B56] Low S. E. Amburgey K. Horstick E. Linsley J. Sprague S. M. Cui W. W. (2011). TRPM7 is required within zebrafish sensory neurons for the activation of touch-evoked escape behaviors. *J. Neurosci.* 31 11633–11644 10.1523/JNEUROSCI.4950-10.2011 21832193 PMC3164782

[B57] Low S. E. Ryan J. Sprague S. M. Hirata H. Cui W. W. Zhou W. (2010). touché Is required for touch-evoked generator potentials within vertebrate sensory neurons. *J. Neurosci.* 30 9359–9367 10.1523/JNEUROSCI.1639-10.2010 20631165 PMC2921932

[B58] Low S. E. Woods I. G. Lachance M. Ryan J. Schier A. F. Saint-Amant L. (2012). Touch responsiveness in zebrafish requires voltage-gated calcium channel 2.1b. *J. Neurophysiol.* 108 148–159 10.1152/jn.00839.2011 22490555 PMC3434603

[B59] Maddatu T. P. Grubb S. C. Bult C. J. Bogue M. A. (2012). Mouse Phenome Database (MPD). *Nucleic Acids Res.* 40 D887–D894 10.1093/nar/gkr1061 22102583 PMC3245053

[B60] Mahabir S. Chatterjee D. Buske C. Gerlai R. (2013). Maturation of shoaling in two zebrafish strains: a behavioral and neurochemical analysis. *Behav. Brain Res.* 247 1–8 10.1016/j.bbr.2013.03.013 23518435 PMC3646909

[B61] Maher A. R. Maglione M. Bagley S. Suttorp M. Hu J. H. Ewing B. (2011). Efficacy and comparative effectiveness of atypical antipsychotic medications for off-label uses in adults: a systematic review and meta-analysis. *JAMA* 306 1359–1369 10.1001/jama.2011.1360 21954480

[B62] Marzullo T. C. Gage G. J. (2012). The SpikerBox: a low cost, open-source bioamplifier for increasing public participation in neuroscience inquiry. *PLoS ONE* 7:e30837 10.1371/journal.pone.0030837 PMC3310049 22470415

[B63] Mathuru A. S. Kibat C. Cheong W. F. Shui G. Wenk M. R. Friedrich R. W. (2012). Chondroitin fragments are odorants that trigger fear behavior in fish. *Curr. Biol.* 22 538–544 10.1016/j.cub.2012.01.061 22365850

[B64] Mojtabai R. Olfson M. (2014). National trends in long-term use of antidepressant medications: results from the U.S. National Health and Nutrition Examination Survey. *J. Clin. Psychiatry* 75 169–177 10.4088/JCP.13m08443 24345349

[B65] Muto A. Ohkura M. Abe G. Nakai J. Kawakami K. (2013). Real-time visualization of neuronal activity during perception. *Curr. Biol.* 23 307–311 10.1016/j.cub.2012.12.040 23375894

[B66] Olfson M. Kroenke K. Wang S. Blanco C. (2014). Trends in office-based mental health care provided by psychiatrists and primary care physicians. *J. Clin. Psychiatry* 75 247–253 10.4088/JCP.13m08834 24717378

[B67] Olson B. D. Sgourdou P. Downes G. B. (2010). Analysis of a zebrafish behavioral mutant reveals a dominant mutation in *atp2a1/SERCA1*. *Genesis* 48 354–361 10.1002/dvg.20631 20533403 PMC2885577

[B68] Pardo-Martin C. Allalou A. Medina J. Eimon P. M. Wählby C. Fatih Yanik M. (2013). High-throughput hyperdimensional vertebrate phenotyping. *Nat. Commun.* 4 1467 10.1038/ncomms2475 PMC3573763 23403568

[B69] Pardo-Martin C. Chang T. Y. Koo B. K. Gilleland C. L. Wasserman S. C. Yanik M. F. (2010a). High-throughput *in vivo* vertebrate screening. *Nat. Methods* 7 634–636 10.1038/nmeth.1481 20639868 PMC2941625

[B70] Pardo-Martin C. Chang T. Y. Koo B. K. Gilleland C. L. Wasserman S. C. Yanik M. F. (2010b). High-throughput *in vivo* vertebrate screening. *Nat Methods* 7 634–636 10.1038/nmeth.1481 20639868 PMC2941625

[B71] Paul S. M. Mytelka D. S. Dunwiddie C. T. Persinger C. C. Munos B. H. Lindborg S. R. (2010). How to improve R&D productivity: the pharmaceutical industry’s grand challenge. *Nat. Rev. Drug Discov.* 9 203–214 20168317 10.1038/nrd3078

[B72] Pérez-Escudero A. de Polavieja G. G. (2011). Collective animal behavior from Bayesian estimation and probability matching. *PLoS Comput. Biol.* 7:e1002282 10.1371/journal.pcbi.1002282 PMC3219619 22125487

[B73] Portugues R. Feierstein C. E. Engert F. Orger M. B. (2014). Whole-brain activity maps reveal stereotyped, distributed networks for visuomotor behavior. *Neuron* 81 1328–1343 10.1016/j.neuron.2014.01.019 24656252 PMC4448760

[B74] Prober D. A. Rihel J. Onah A. A. Sung R. J. Schier A. F. (2006). Hypocretin/orexin overexpression induces an insomnia-like phenotype in zebrafish. *J. Neurosci.* 26 13400–13410 10.1523/JNEUROSCI.4332-06.2006 17182791 PMC6675014

[B75] Prober D. A. Zimmerman S. Myers B. R. McDermott B. M. Jr. Kim S. H. Caron S. (2008). Zebrafish TRPA1 channels are required for chemosensation but not for thermosensation or mechanosensory hair cell function. *J. Neurosci.* 28 10102–10110 10.1523/JNEUROSCI.2740-08.2008 18829968 PMC2728686

[B76] Qin M. Wong A. Seguin D. Gerlai R. (2014). Induction of social behavior in zebrafish: live versus computer animated fish as stimuli. *Zebrafish* 11 185–197 10.1089/zeb.2013.0969 24575942 PMC4050712

[B77] Rihel J. Prober D. A. Arvanites A. Lam K. Zimmerman S. Jang S. (2010). Zebrafish behavioral profiling links drugs to biological targets and rest/wake regulation. *Science* 327 348–351 10.1126/science.1183090 20075256 PMC2830481

[B78] Robinson K. S. Stewart A. M. Cachat J. Landsman S. Gebhardt M. Kalueff A. V. (2013). Psychopharmacological effects of acute exposure to kynurenic acid (KYNA) in zebrafish. *Pharmacol. Biochem. Behav.* 108 54–60 10.1016/j.pbb.2013.04.002 23583441

[B79] Satou C. Kimura Y. Hirata H. Suster M. L. Kawakami K. Higashijima S. (2013). Transgenic tools to characterize neuronal properties of discrete populations of zebrafish neurons. *Development* 140 3927–3931 10.1242/dev.099531 23946442

[B80] Schadt E. E. Friend S. H. Shaywitz D. A. (2009). A network view of disease and compound screening. *Nat. Rev. Drug Discov.* 8 286–295 10.1038/nrd2826 19337271

[B81] Sneader W. (1996). *Drug Prototypes and Their Exploitation.* Hoboken: Wiley

[B82] Sneader W. (2005). *Drug Discovery.* Hoboken: John Wiley & Sons 10.1002/0470015535

[B83] Speedie N. Gerlai R. (2008). Alarm substance induced behavioral responses in zebrafish (*Danio rerio*). *Behav. Brain Res.* 188 168–177 10.1016/j.bbr.2007.10.031 18054804 PMC2715551

[B84] Stewart A. Gaikwad S. Kyzar E. Green J. Roth A. Kalueff A. V. (2012). Modeling anxiety using adult zebrafish: a conceptual review. *Neuropharmacology* 62 135–143 10.1016/j.neuropharm.2011.07.037 21843537 PMC3195883

[B85] Stewart A. Riehl R. Wong K. Green J. Cosgrove J. Vollmer K. (2011). Behavioral effects of MDMA (‘ecstasy’) on adult zebrafish. *Behav. Pharmacol.* 22 275–280 10.1097/FBP.0b013e328345f758 21522057 PMC3083639

[B86] Stewart A. M. Kalueff A. V. (2014). The behavioral effects of acute Δ^9^-tetrahydrocannabinol and heroin (diacetylmorphine) exposure in adult zebrafish. *Brain Res.* 1543 109–119 10.1016/j.brainres.2013.11.002 24216135

[B87] Szobota S. Gorostiza P. Del Bene F. Wyart C. Fortin D. L. Kolstad K. D. (2007). Remote control of neuronal activity with a light-gated glutamate receptor. *Neuron* 54 535–545 10.1016/j.neuron.2007.05.010 17521567

[B88] Tecott L. H. Nestler E. J. (2004). Neurobehavioral assessment in the information age. *Nat. Neurosci.* 7 462–466 10.1038/nn1225 15114359

[B89] U.S. Food and Drug Administration. (2014). *FDALabel.* Available at: http://www.fda.gov/ScienceResearch/BioinformaticsTools/ucm289739.htm [accessed April 13 2014]

[B90] Valente A. Huang K. H. Portugues R. Engert F. (2012). Ontogeny of classical and operant learning behaviors in zebrafish. *Learn. Mem.* 19 170–177 10.1101/lm.025668.112 22434824 PMC3312620

[B91] Williams L. R. Wong K. Stewart A. Suciu C. Gaikwad S. Wu N. (2012). Behavioral and physiological effects of RDX on adult zebrafish. *Comp. Biochem. Physiol. C Toxicol. Pharmacol.* 155 33–38 21382508 10.1016/j.cbpc.2011.02.010

[B92] Wittmann C. Reischl M. Shah A. H. Mikut R. Liebel U. Grabher C. (2012). Facilitating drug discovery: an automated high-content inflammation assay in zebrafish. *J. Vis. Exp.* 65 e4203. 10.3791/4203 PMC3476412 22825322

[B93] Wolman M. A. Jain R. A. Liss L. Granato M. (2011). Chemical modulation of memory formation in larval zebrafish. *Proc. Natl. Acad. Sci. U.S.A.* 108 15468–15473 10.1073/pnas.1107156108 21876167 PMC3174630

[B94] Wyart C. Del Bene F. Warp E. Scott E. K. Trauner D. Baier H. (2009). Optogenetic dissection of a behavioural module in the vertebrate spinal cord. *Nature* 461 407–410 10.1038/nature08323 19759620 PMC2770190

[B95] Yokogawa T. Marin W. Faraco J. Pézeron G. Appelbaum L. Zhang J. (2007). Characterization of sleep in zebrafish and insomnia in hypocretin receptor mutants. *PLoS Biol.* 5:e277 10.1371/journal.pbio.0050277 PMC2020497 17941721

[B96] Zhdanova I. V. (2006). Sleep in zebrafish. *Zebrafish* 3 215–226 10.1089/zeb.2006.3.215 18248262

